# Role of Cell-Cell Junctions in Oesophageal Squamous Cell Carcinoma

**DOI:** 10.3390/biom12101378

**Published:** 2022-09-26

**Authors:** Qian-Rui Xu, Xiao-Hui Du, Ting-Ting Huang, Yu-Chun Zheng, Yu-Ling Li, Dan-Yi Huang, Hao-Qiang Dai, En-Min Li, Wang-Kai Fang

**Affiliations:** Department of Biochemistry and Molecular Biology, Shantou University Medical College, Shantou 515041, China

**Keywords:** adherens junction, tight junction, desmosome, gap junction, epithelial–mesenchymal transition, cancer metastasis, oesophageal squamous cell cancer

## Abstract

Cell–cell junctions comprise various structures, including adherens junctions, tight junctions, desmosomes, and gap junctions. They link cells to each other in tissues and regulate tissue homeostasis in critical cellular processes. Recent advances in cell–cell junction research have led to critical discoveries. Cell–cell adhesion components are important for the invasion and metastasis of tumour cells, which are not only related to cell–cell adhesion changes, but they are also involved in critical molecular signal pathways. They are of great significance, especially given that relevant molecular mechanisms are being discovered, there are an increasing number of emerging biomarkers, targeted therapies are becoming a future therapeutic concern, and there is an increased number of therapeutic agents undergoing clinical trials. Oesophageal squamous cell carcinoma (ESCC), the most common histological subtype of oesophageal cancer, is one of the most common cancers to affect epithelial tissue. ESCC progression is accompanied by the abnormal expression or localisation of components at cell–cell junctions. This review will discuss the recent scientific developments related to the molecules at cell–cell junctions and their role in ESCC to offer valuable insights for readers, provide a global view of the relationships between position, construction, and function, and give a reference for future mechanistic studies, diagnoses, and therapeutic developments.

## 1. Overview

Epithelial cells exhibit several types of cell–cell junctions that can be classified into adherens junctions, tight junctions, desmosomes, and gap junctions. Cell–cell junctions play an essential role in the maintenance of epithelial homeostasis. During various physiological processes, such as tissue development, wound healing, or tumorigenesis, cellular junctions are reorganised to allow the release or the incorporation of individual cells. Abnormalities in the organisation of these junctions are common in genetic and metabolic disorders of the epithelia [1]. Their compositions are dynamic and regulated by complex protein networks. The imbalance of these networks, caused by oncogenic proteins or pathogens, results in barrier breakdown and eventually leads to cancer; this may be due to the disorder between the partial dismantling and re-establishment of cell–cell contact [2]. Deregulation of molecules in the junctions contributes to tumour metastasis. Loss of cell–cell contact also facilitates the migration of tumour cells to distal sites [3].

Here, we focus on oesophageal squamous cell carcinoma (ESCC), the most common histological subtype of oesophageal cancer. Oesophageal cancer ranks seventh in terms of incidence (604,000 new cases) and sixth in mortality overall (544,000 deaths) [4]. Studies have shown that many components of these cell–cell junctions are up-regulated or down-regulated in ESCC; for example, E-Cadherin, α -Catenin, DSC2, and claudins in the plasma membranes of ESCC cells decrease compared with their levels in normal tissues [5,6]. Loss of regulation results in the onset of disease progression and cancer. For example, the absence of E-Cadherin contributes to the development and metastasis of tumours [7]. Decreased DSC2 and E-Cadherin are found to be connected with low survival rates in ESCC patients [8]. The low expression of Claudin-4 in ESCC is associated with poor prognosis [9]. These expression changes are often accompanied by changes in localisation.

Among the four types of cell–cell junctions, adherens, and tight junctions are often discussed together because their components can interact and modulate each other’s activities closely. For example, there is reciprocal regulation between E-Cadherin in adherens junctions and Claudin-7 in tight junctions [10,11,12]. Moreover, there are other components such as plakoglobin (PG), which is a major cytoplasmic component of both desmosomes and adherens junctions; therefore, it is evident that these junction structures are not independent [13]. The E-Cadherin and N-Cadherin switch during the epithelial-to-mesenchymal transition (EMT) is regulated by a complex network of signalling pathways and transcription factors, which are widely studied in different cancers [14]. In ESCC, upregulating mesenchymal markers such as N-Cadherin, vitamins, and β-catenin are related to vasculogenic mimicry formation, invasion, and metastasis [15,16,17]. Molecules altering the EMT proteins can also predict poor prognoses and low survival rates of ESCC [18].

Generally, the current studies on intercellular junctions and ESCC have been limited to specific adhesion molecules. There has been no systematic summary based on the four types of cell–cell junctions, which would be more conducive for readers as it would allow them to roundly view the relationships between position, construction, and function of the different types of junction structures. 

**Figure 1:**
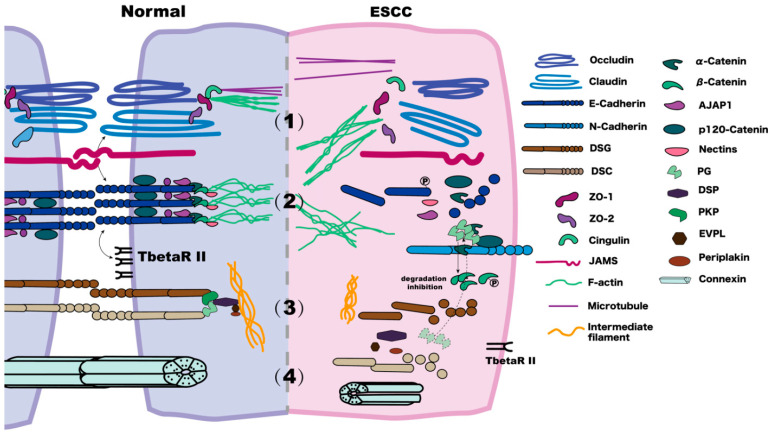
Comparison of normal and ESCC cell–cell junctions. (1) In the tight junction of a normal cell, the N-terminus of ZO interacts with claudins, JAMs, and occludin, and the C-terminus interacts with F-actin. Cingulin connects with the microtubules. In ESCC, membrane claudins, occludins, and ZOs decrease on the membrane. (2) In adherens junctions, the cytoplasmic domain of E-Cadherin forms a ternary complex with β-Catenin and α-Catenin, bound by p120-Catenin. α-Catenin binds to F-actin, linking Catenin-based complexes to the actin cytoskeleton. In ESCC, E-Cadherin is reduced via internalisation and is phosphorated; the expression of TbetaRII decreases. The ESCC metastasis often refers to co-localisation among β-Catenin, PG, and E-Cadherin. There is an E- to N-Cadherin expression change. There is a decrease of α-Catenin and p120-Catenin and an increase in the levels and phosphorylation of β-Catenin. (3) A desmosome consists of DSCs, DSGs, PG, Pkps, DSP, EVPL, and periplakin. In the normal cell, the primary function of DSCs and DSGs is to mediate the assembly of the desmosome and E-Cadherin maturation. In ESCC, under low DSC2 conditions, keratin intermediate filaments retract. There is a modest expression of DSG1, but DSG3 has unclear current research results. PG, which normally has a role in “cross-talking” between adherens junctions and desmosomes, may increase and compete with β-Catenin when binding to E-Cadherin, thus leading to the release of β-Catenin. (4) Gap junctions in normal tissues are mainly hexameric complexes of Cx proteins. In ESCC with a low degree of malignancy, the expression of Cx26 is absent, and Cx43 is decreased.

We reviewed 160 published papers related to this topic and identified some possible mechanisms to help convert the discovery work from bench to bedside. This review mainly summarises the changes of the components related to the tight junctions, adherens junctions, desmosomes, and gap junctions in ESCC (Figure 1), as well as some cross-talks between them, thus providing a reference for future studies on their mechanisms, diagnosis of ESCC, and therapeutic developments. 

## 2. Tight Junctions

Tight junctions, located at the apical region of cell–cell contacts, have two main functions, serving as both a “fence” and “gate”. As a “fence”, the tight junction restricts the exchange of membrane components between the apical and basolateral cell surface domains. As a “gate”, it regulates paracellular free diffusion of ions and molecules across the epithelial cell sheet, thus acting as an osmotic barrier for the tissues and different compartments of the body [19]. The molecular composition of tight junctions has been widely studied, and it mainly involves the transmembrane claudins, and the adapter protein zonula occludens (ZO), which is connected to claudins in the cytoplasm. In addition, other components, such as tight junction-associated MARVEL proteins (TAMP) (occludin, tricellulin, marvelD3), and junction adhesion molecules (JAMs), are also located around the tight junctions [20].

Claudin is a four transmembrane protein whose positively and negatively charged residues in the first extracellular domain determine the permeability of ions with different sizes and charges. For tight junctions to form in epithelial cells, claudins polymerize linearly to generate tight junction strands in the most apical parts of the lateral membranes [21], and the width of tight junction strands, revealed by freeze-fracture electron microscopy, suggests that two linear claudin polymers associate with each other in an antiparallel double row fashion [22]. 

Occludin plays a regulatory role in tight junctions and forms a platform for the signal transduction process. It has been determined that a variety of stimuli can regulate the structure and function of tight junctions through occludin, including growth factors (such as hepatocyte growth factor), inflammatory factors (such as interleukin), TNF- α, IFN- γ, and so on; however, tight junctions can form normally without occludin, meaning that the mechanism is undetermined. JAMs are members of an immunoglobulin superfamily that interact with tight junctions and adherens junction proteins to regulate barrier function, cell migration, and cell proliferation [1].

The Zos include ZO-1, ZO-2, and ZO-3. Zos contain a variety of domains, and they interact with various proteins, such as claudins, F-actin, adhesive connection proteins, such as α-Catenin and afadin, and signal proteins [20]. The N-terminus of ZO-1 consists of PSD95, DlgA, and ZO-1 (PDZ) homology domains, an SRC homology 3 (SH3) domain, and a guanylate kinase (GUK) homology domain that interacts with different proteins. The PDZ1 domain interacts with claudins, PDZ3 interacts with JAMs, GUK interacts with occludins, and SH3 interacts with the ZO-1-associated nucleic acid binding protein, (ZONAB)/heat shock 70 kD protein 4 (HSP70RY). ZONAB is a proliferation-regulating transcription factor localised in tight junctions and nuclei. ZONAB enhances proliferation in the nucleus, but ZONAB inhibits proliferation by binding to ZO-1 at the tight junctions [19]. The C-terminus of ZO-1 interacts with F-actin. The function of the interaction between ZO-1and F-actin is not clear; however, it has been predicted that ZO-1 regulates the majority of cytoskeletal tension, and ZO-1 deletion could lead to dysregulated and excessive cytoskeletal tension that would ultimately cause epithelial apical specialisation abnormalities [19,23]. 

### 2.1. Effects and Possible Mechanisms of the Abnormal Expression of Claudins and Occludins in ESCC

In multiple tumour types, tumour progression is related to abnormal changes in tight junctions. Claudins 1, 4, and 7 are expressed on the cell membrane of normal human oesophageal keratinocytes; however, their typical localisation is reduced or lost at the ESCC cell membrane, and instead, are wrongly redistributed to the cytoplasm and perinuclear region [24]. 

Low expression of claudin-1 on the membrane is associated with recurrence and poor prognosis of ESCC [25]. Mechanistically, it is shown that this could occur through the reduced non-metastatic protein 23-H1 (Nm23H1, a metastasis suppressor), thus resulting in the activation of the Akt signalling pathway and enhanced invasiveness of ESCC cells [26]. Another study found that claudin-1 become abundant in the ESCC nucleus, triggering autophagy through the AMPK/STAT1/ULK1 signalling pathway to promote proliferation and metastasis [27]. As for claudin-4, it expresses at low levels in ESCC and may relate to promoter methylation. Low expression of claudin-4 is associated with poor differentiation, depth of invasion, and lymph node metastasis. It also negatively affects disease-free and overall survival [9] as an independent risk factor for recurrence [28]. Decreased claudin-7 localisation at the invasive front is observed in oesophageal cancer, and this phenomenon relates to the depth of invasion, stage, lymphatic vessel invasion, and lymph node metastasis [24]. Beyond these three claudins, claudin-18 is often lost in tumours, inducing gastric cancer, subsequently affecting cytokines and stemness; additionally, it might also relate to Wnt-signaling pathways in other tumour types. The role of claudin-18 in ESCC is worthy of further research [29].

Studies show that Snail expression is related to high levels of vimentin and low levels of E-Cadherin, claudin-1, claudin-7, and occludin. High expressions of Snail at the invasive front of the oesophageal tumours are associated with a high incidence of tumour invasions in lymph and venous vessels, lymph node metastasis, and the tumour clinicopathological stage [30]. Snail, a zinc-finger transcription inhibitor, responds to different microenvironments, and acts as a molecular switch for EMT, when promoting tumour metastasis [31]. The primary mechanism is as follows: Snail binds to the E-box in the E-Cadherin promoter, thereby inhibiting the transcription of E-Cadherin (discussed below) [32]. A similar mechanism results in the loss of claudin-1, claudin-7, and occludin [33,34]. Thus, Snail is critical to the abnormally tight junctions in ESCC. Recently, a study of 10 human SCC cell lines showed that Snail-induced claudin-11 prompts collective migration for tumour progression. The authors proposed the novel idea that claudin-11 contributes to the maintenance of cell–cell contacts during tumour metastasis to enhance metastatic efficiency. This extends the current understanding of EMT-mediated cellular migration via a non-individual type of movement, and it helps us notice more about the role of cell–cell junction proteins in terms of increasing metastatic efficiency [35].

In other cancers, there is evidence that the interference of claudin is associated with EGFR signalling. For example, claudin-2 is upregulated in colon cancer to promote tumour cell proliferation, and this upregulation depends on EGFR signalling and its downstream pathways. In epidermal growth factor mutant waved-2 mice, which have reduced EGFR tyrosine kinase activity, the expression of claudin-2 strongly decreases [36]. As a new type of approach, targeted therapies have been confirmed to play an essential role in treating oesophageal cancer, such as cetuximab targeting the EGFR. Does this relate to claudins? Answering this question may help with therapeutic developments. 

### 2.2. ZO Family Roles and Alterations in ESCC

In addition to the two critical transmembrane proteins (claudin and occludin), ZO, an adapter protein, links to membrane proteins (such as claudin and occludin) and the actin cytoskeleton, which also shows changes in ESCC. Normally, ZO-1 is co-localised with PAR-3 (partitioning-defective-3) at sites of cell–cell contact. In normal epithelial cells, PAR-3, PAR-6, and aPKC comprise a conserved protein complex that regulates cell polarisation and participates in the formation of tight junctions between epithelial cells and other cells [37,38]. A study found a homozygous deletion of the gene encoding PAR-3 and copy number loss of PAR-3 in ESCC, which was associated with positive lymph node metastasis and poor differentiation of primary ESCC. In ESCC cells lacking the gene encoding PAR-3, ZO-1 is barely detected. Exogenous expression of PAR-3 enhances the recruitment of ZO-1 to sites of cell–cell contact without affecting the expression level of ZO-1. Conversely, the knockdown of PAR-3 inhibits the localisation of ZO-1 to points of cell-cell contact. These indicated that the deletion and reduced expression of PARD3 may be a novel mechanism in the progression of ESCC [39].

## 3. Adherens Junctions

The adherens junction regulates the organisation of the cytoplasmic actin cytoskeleton and establishes a hub for cell signalling [40]. It is characterised by two cell membranes, spaced about 10–20 nm apart, and occupied by rod-like molecules bridging the plasma membranes [40,41]. Classical cadherins, such as E-Cadherin, are the primary transmembrane glycoproteins constituting adherens junctions, and they contain five extracellular cadherin repeat domains that engage in Ca2+-dependent trans binding to a cadherin on the opposing cell surface [42]. The cytoplasmic domain of E-Cadherin forms a ternary complex with β-Catenin (a member of the armadillo protein family) and α-Catenin, the former of which is mechanically attached to the actin cytoskeleton [1] after α-Catenin binds to F-actin in a force-dependent manner [43]. p120-Catenin also binds to the ternary complex and regulates the lifetime of E-Cadherin on the plasma membrane [44]. Another immunoglobulin-like adhesion molecule, Nectin, forms calcium-independent intercellular adhesions in adherens junctions [45]. Nectin binds afadin, which also binds α-Catenin [46] and ZO-1 [47] in order to link Catenin-based complexes to the actin cytoskeleton; thus, adherens junctions are closely related to the tight junctions discussed above.

### 3.1. Abnormal Expression of E-Cadherin in ESCC Cells and Possible Mechanisms

Studies found that abnormalities in E-Cadherin localisation and expression level play an essential role in the invasion and metastasis of ESCC [48,49]. E-Cadherin may serve as a biomarker for precancerous lesions and a predictor of lymph node metastasis and prognosis [49,50,51,52]. The abnormities can be summarised in the following two ways. 

The first is abnormal cellular localisation. E-Cadherin in normal epithelial tissue (non-metaplastic and non-dysplastic metaplastic tissue) shows a high expression on the surface membrane. In contrast, ESCC cells are characterised by the cytoplasmic localisation of E-Cadherin and they are associated with poor differentiation. This phenomenon may be caused by reduced glycosylation or protein truncation of E-Cadherin [50]. 

The second is the reduction or absence of expression. A study found that E-Cadherin expression is associated with the differentiation of ESCC, and it may serve as a biomarker of differentiation. E-Cadherin is highly expressed in the normal oesophageal squamous epithelium, but it is expressed to a lesser extent, or absent, in poorly differentiated ESCC [53]. 

Moreover, the abnormal expression and localisation of E-Cadherin enhances the migration and invasion ability of ESCC cells, not only as it leads to the loss of cell polarity and the derangement of normal tissue architecture to reduce adhesion, but also as it maintains tumour basal cells in the basal cell layer of ESCC tissue [54]. There is a lack of coordination between E-Cadherin and TbetaRII (TGFβ receptor II) in most ESCC samples, as TbetaRII-mediated cell signalling depends on intact E-Cadherin function [55]. The up-regulation of TbetaRII contributes to maintain a basal-like phenotype of tumour basal cells in ESCC, and it correlates with enhanced migration and poor survival rates [54].

The decreased expression often occurs at the transcriptional level. The 5′ CpG island of the E-Cadherin gene is methylated in most ESCC samples and is an important reason for reducing or inhibiting transcription and translation [56]. The DNA methylation level may be a significant predictor of prognosis, and it is involved in the progression of ESCC [57]. EphA3 expression in ESCC tissues and cell lines also decreases due to DNA methylation. EphA3 is one of the Eph receptor tyrosine kinases critical for cell–cell communication during normal and oncogenic development. Overexpression of EphA3 in ESCC cells can induce up-regulation of ZO-1 and E-Cadherin at the transcriptional level and inhibit cell migration and invasion via the Rho GTPase signalling pathway [58]. This suggests that EphA3 has a tumour-suppressive effect on ESCC; however, the specific downstream pathway of Rho GTPase, affected by EphA3, is not well characterised.

MicroRNA25 (miR-25) is also involved in the regulation of E-Cadherin expression. E-Cadherin is a direct target of miR-25 and there is a negative correlation between miR-25 and E-Cadherin in ESCC tumour tissues. In ESCC, the high expression of miR-25 inhibits E-Cadherin expression, which is crucial for mediating EMT, thus leading to the migration and invasion of ESCC [59].

Members of the p53 gene family are also involved in the regulation of E-Cadherin expression. p53 gene mutation is the most frequent genetic alteration in typical squamous cell carcinoma of the oesophagus [60]. In about half of all human cancers, the p53 gene is either lost or mutated [61]. A study on prostate cancer found that p53 mutation or downregulation in cancer cells significantly reduced the expression of the RNA Binding Motif Protein 25 (RBM25). This inhibited the expression of angiomotin-like 1-derived circRNA (circAMOTL1L), a miR-193a-5p sponge, thereby enabling the miR-193a-5p-mediated repression of the protocadherin α cluster (a subset of the cadherin superfamily members), thus down-regulating E-Cadherin and inducing EMT [62]. This has not yet been shown for ESCC. 

p63, a member of the p53 family, binds to a specific enhancer region of the zinc finger protein 185 (ZNF185), along with a LIM domain gene promoter, in order to up-regulate ZNF185 expression for sustaining epithelial differentiation and regulating intercellular adhesion during epithelial development. ZNF185 physically interacts with E-Cadherin on the membrane to maintain epithelial integrity. The absence of ZNF185 in ESCC, especially in poorly differentiated cells, suggests that p63 downregulates E-Cadherin via ZNF185 in cancer. Similar studies in ESCC reinforce that p63 is a crucial gene for maintaining epithelial tissue integrity and supporting the deregulation of cell–cell adhesion programming, which plays a critical role in carcinoma development [63].

Researchers also found that venous fluid shear stress can affect E-Cadherin in ESCC cells. Venous shear (shear rate, 200/s) induces the internalisation of E-Cadherin in metastatic ESCC tumour cells (OC-1 tumour cell line) by activating the Src pathway and phosphorylating tyrosine residues in the short intracytoplasmic tail of E-Cadherin [64]. P120-Catenin is also an Src substrate [65], and it also affects cadherin adhesiveness [66]; however, this has not been proven in ESCC. Clinically, high expression levels of E-Cadherin, and the preservation of membranous p120-Catenin, are positively correlated with tumour differentiation.p120-Catenin expression is correlated with E-Cadherin expression and lymph node metastasis [67]. p120-Catenin is required for Src-induced oncogenic transformation and it provides a potential target for future therapeutic interventions. 

However, a study about ovarian cancer found that E-Cadherin knockout decreases the expression of β-Catenin and its transcriptional target cyclin D1, and the migratory ability and the cellular response to Rho GTPase inhibitors are also inhibited. Thus, even if the expression of E-Cadherin decreases, it might still play an essential role in regulating tumour development [68]; for instance, E-Cadherin plays an integral role in generating front/back polarity and keeping directional movement during collective cell migration. Knockdown of E-Cadherin results in the disorientation of collective migration and decreased migration speed [69].

We mentioned that adherens junctions are closely related to tight junctions; E-Cadherin and claudin-7, a component of tight junctions, display synergy in ESCC cells. Decreased expression of claudin-7 leads to the reduced expression of E-Cadherin, increased cell growth, and enhanced invasion in a three-dimensional matrix. In normal oesophageal epithelial cells, claudin-7 is limited to the membrane of differentiated keratinocytes, whereas in ESCC samples, claudin-7 is often lost or restricted in the cytoplasm [12]. The mechanism could be due to the binding of Snail to the E-box in the E-Cadherin promoter mentioned in Section 2.1.

### 3.2. Possible Effects and Mechanisms of α- and β-Catenin on the Invasiveness and Metastasis of ESCC

Decreased α-Catenin expression is associated with poor prognosis in ESCC [70] and it has an even greater association with aggressive phenotypes and lymph node metastasis than E-Cadherin [71]. Tumour induction in rats shows that changes in α-Catenin cell localisation might provide a clue to the tumour progression of oesophageal carcinoma [72]. Since the abovementioned studies did not focus on ESCC, but extensively studied oesophageal carcinoma, this still needs further validation.

β-Catenin is a multifunctional protein involved in cell–cell adhesion and signal transduction. It is found abnormally accumulated in the cytoplasm and nucleus, and it is phosphorylated in ESCC [73,74]. Studies have shown that the over-expression of FRAT1 and EB1 activates the β-Catenin/TCF pathway, resulting in the nuclear accumulation of β-Catenin [75,76]. The glycogen synthase kinase-3β (GSK-3β) pathway is seen as another pathway associated with β-Catenin expression. Research has shown that the expression of migfilin (filamin-binding LIM protein 1) in ESCC with lymph node metastases was lower than the mean expression in ESCC without metastases. Low migfilin can activate β-Catenin–mediated transcription, increase β-Catenin protein levels by preventing proteasomal degradation, and inhibit GSK3β-mediated degradation of β-Catenin [77]. We mentioned that Snail is a major regulator of PAR-3 protein expression. Upregulated Snail inhibits PAR-3, then inhibits the localisation of ZO-1 at cell–cell contact points; this is because the ZO-1 protein is likely regulated by the GSK-3β/Snail/Par3/ZO-1 axis [78]. 

Phosphorylation of β-Catenin is associated with Aurora-A (encoding a serine/threonine-protein kinase) and an epidermal growth factor receptor (EGFR). Studies revealed that Aurora-A kinase can inhibit β-Catenin degradation and promote β-Catenin dissociation from cell–cell contacts, nuclear translocation, and TCF/LEF-1 transcriptional activity through phosphorylating β-Catenin at Ser552 and Ser675, thus resulting in the enhancement of invasion and metastasis in ESCC. This provides new insight into the role of Aurora-A in tumour promotion via the activity of the β-Catenin pathway [79]. The epidermal growth factor (EGF) in oesophageal cancer cells can activate EGFR [80] to phosphorylate β-Catenin, subsequently resulting in a disrupted bond between β-Catenin and α-Catenin [81,82]; therefore, the connection between E-Cadherin and the actin cytoskeleton is disrupted [83], resulting in a more aggressive phenotype [80]. 

### 3.3. Potential of N-Cadherin as a Biomarker of ESCC

N-Cadherin, a classic cadherin family member, is primarily expressed in nerve, muscle, and mesenchymal cells, and it also mediates cell–cell adherens junctions. E- and N-Cadherin mostly have mutually exclusive expression patterns, with E-Cadherin being expressed primarily in epithelial cells; however, in the context of EMT, N-Cadherin can induce changes in the biological behaviour of cells in favour of the migratory phenotype, and the ability of tumour cells to alter their cadherin expression profiles, such as transitioning from E- to N-cadherin, is critical for malignant progression [84,85,86]. There is increased N-Cadherin in ESCC [16]. During cancer cell migration, E/N-Cadherin-based cell–cell junctions sharpen the cell boundaries, resulting in a relatively weak (compared with E-Cadherin) adherens junction between cells to promote the effective dissemination of cells in the process of collective migration [87].

**Figure 2:**
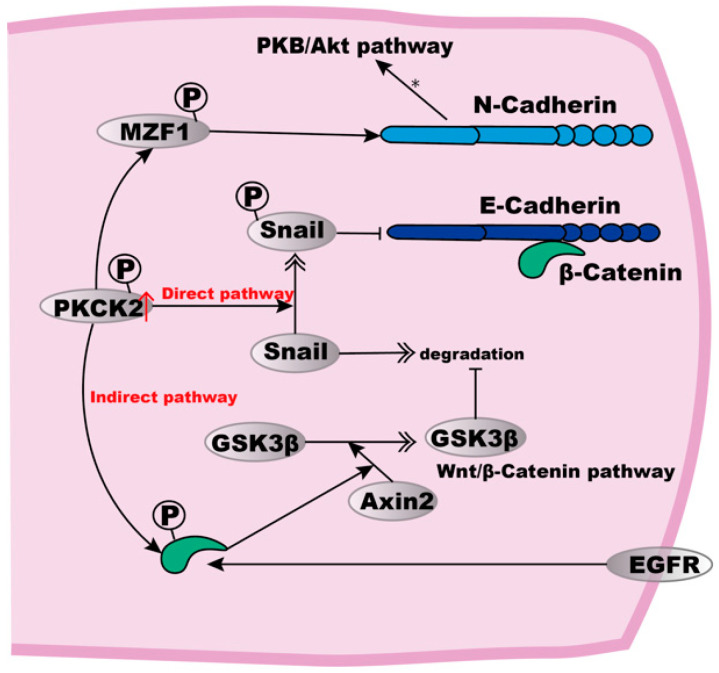
E- to N-Cadherin transition in ESCC cells. High expression of PKCK2 can induce E- to N-Cadherin transition. In a direct way, PKCK2 phosphorylation stabilises Snail, which then inhibits the expression of E-Cadherin. The indirect pathway involves the PKCK2 phosphorylation to cause β-Catenin-mediated Axin2 expression, thus resulting in Axin2-mediated GSK3β translocation from the nucleus to the cytosol where GSK3β inhibits Snail degradation. For the up-regulation of N-Cadherin, MZF1 may be phosphorylated to up-regulate N-Cadherin expression, subsequently activating the PKB/Akt signalling pathway. In addition, EGFR can also phosphorylate β-Catenin, leading to the dissociation of E-Cadherin from the actin cytoskeleton.

Studies have found that the high activity of protein kinase casein kinase 2 (PKCK2) in ESCC cells can induce E- to N-Cadherin transformation by directly or indirectly stabilising Snail proteins, thus leading to the down-regulation of E-Cadherin [88,89] (Figure 2). Snail can be directly activated by phosphorylation at Ser92, or indirectly by the phosphorylation of β-Catenin at Thr393, which then up-regulates the β-Catenin-mediated expression of Axin2, a protein involved in β-Catenin stabilisation and the Wnt pathway [89]. At the same time, Axin2 mediates a GSK3β export from the nucleus to prevent GSK3β-mediated Snail degradation [88,90]. Combined with the studies above, we find that GSK3β inhibits the degradation of both β-Catenin and Snail, thus influencing many components such as ZO-1 and activities in the adherens junctions, thus resulting in stronger intercellular junctions, which also indicates the critical role of the related pathways in ESCC. 

PKCK2 phosphorylates target several proteins to regulate their function [91,92,93] or the expression of downstream genes [90,93]. For the up-regulation of N-Cadherin, it is possible that myeloid zinc finger-1 (MZF1), a known transcription factor required for N-Cadherin gene expression, could be a substrate of PKCK2, and may thus become stabilised to upregulate N-Cadherin expression [94]. Subsequently, the increased expression of N-Cadherin can activate the PKB/Akt signalling pathway, thus resulting in resistance to both hypoxia and anoikis [88]. 

### 3.4. Two Possible Adhesion Proteins Affecting Migration and Invasion of ESCC Cells

It is unknown whether cadherin-23 (Cdh23) and protocadherin 10 (PCDH10) are involved with adherens junctions, but they are involved with intercellular junctions, and they are associated with the migration and invasion of ESCC cells. 

Cdh23 is an atypical member of the cadherin superfamily with a distinctly long extracellular domain [95]. Cdh23 is down-regulated in ESCC cells through promoter methylation. In addition, there is a significant negative correlation between Cdh23 expression and ESCC cell migration [96]. The PCDH10 gene belongs to the protocadherin gene family, a subfamily of the cadherin superfamily. Research has shown that the ectopic expression of PCDH10 strongly inhibits tumour cell growth, migration, invasion, and colony formation. Transcriptional silencing and promoter methylation of both alleles were often found in oesophageal carcinoma cells [97]. These results suggest that PCDH10 expression in ESCC cells may also be altered to affect the biological function of ESCC cells. PCDH10 is frequently inactivated in epigenetic processes in various cancers, suggesting that it may act as a tumour suppressor gene [97]; however, the specific mechanism by which cadherin-23 and PCDH10 affect cancer cells remains unclear.

### 3.5. ESCC and AJAP1

Adherens junctions-associated protein-1 (AJAP1) is a type 1 transmembrane protein that is located and interacts with the E-Cadherin–Catenin complex, which is frequently lost or epigenetically silenced in ESCC cells because of promoter hypermethylation in polarised epithelial cells. Patients with down-regulated AJAP1 transcription are more likely to experience shorter overall and disease-free survival. Multivariate analysis of disease-free survival identified down-regulated AJAP1 transcription as an independent prognostic factor in ESCC [98]. Studies in many cancers found that the abnormal expression of AJAP1 is related to cell migration, invasion, increased tumour growth, and changes in tumour vascularisation, thus suggesting that AJAP1 may be a tumour suppressor [99]. The role of AJAP1 in ESCC progression needs further study.

## 4. Desmosomes

The desmosome is located under the adhesion belt, a speckled anchoring joint among cells. Its principal function is to anchor cytoskeletal keratin intermediate filaments to the cell membrane [100]. Acting as the main intercellular junctions of epithelial cells, desmosomal components can be classified into three types according to gene and function: desmosomal cadherins, armadillo proteins, and plakin proteins [101]. Desmosomal cadherins, which have received the most focus in ESCC research [102,103], are transmembrane glycoproteins that rely on calcium for adhesion, and they consist of desmocollins (DSC1-3) and desmogleins (DSG1-4). Their primary function is to mediate the assembly of desmosomes and E-Cadherin maturation [104]. DSC2, the most widely distributed form of DSCs [105,106], plays a role in the interaction between plaque proteins and intermediate filaments to mediate cell–cell adhesion, and it may also be involved in epidermal cell localisation [107]. For other components of the desmosome, the armadillo family includes plakoglobin (PG, also known as γ-Catenin) and plakophilins (Pkps), and the plakin family has desmoplakin (DSP) and envoplakin (EVPL). DSP connects with intermediate filaments. 

### 4.1. DSC2 Deficiency in ESCC and Its Mechanism

Loss of desmosomes in cancer cells is related to cell migration and reduced adhesion; therefore, it contributes to ESCC invasion and metastasis. In 2010, Fang and his team examined 308 ESCC specimens and found that DSC2 is expressed at lower levels and becomes distributed abnormally in tumours. Moreover, the mRNA and protein levels of two DSC2 isoforms (DSC2a and DSC2b) are lower, and their proteins become redistributed from the membrane to the cytoplasm [108]. This causes keratin intermediate filaments to retract, and it also causes a filamentous (F)-Actin rearrangement in ESCC [109]. This also suggests that the overexpression of DSC2 can inhibit the proliferation and metastasis of ESCC [8].

In terms of a molecular mechanism, DSC2 is the downstream target of microRNA25 (miR-25). In 2013, research showed that miR-25 is highly expressed in ESCC and binds to the 3′UTR of DSC2 mRNA, which causes the down-regulation of DSC2. In these cells, miR25 can also affect the E-Cadherin mRNA, resulting in tumour invasion and metastasis, which is consistent with what occurs in E-cadherin, as previously mentioned [8,59]. Since p53 acts as an upstream for miR25, the authors again emphasised that the manipulation of associated upstream miR-25 regulatory molecules, such as p53, may prevent ESCC development in high-risk patients, which also corresponds with the research on E-Cadherin; this is summarised above in 3.1, which details how the p53 gene family can up-regulate E-Cadherin expression [60]. DSC2 may link to other cell–cell junction components. ESCC metastasis involves the co-localisation of β-Catenin, PG, and E-Cadherin. The down-regulation of DSC2 in ESCC results in increased free PG, which can then compete with the β-Catenin in E-Cadherin/β-Catenin complexes, thus causing an increase in β-Catenin and enabling β-Catenin nuclear translocation and induction of transcriptional activity, which subsequently leads to invasion-associated gene expression and ESCC invasion; therefore, it is not hard to understand why the overexpression of DSC2 inhibits the proliferation and metastasis of ESCC. When DSC2 expression is restored in ESCC cells, β-Catenin re-combines with E-Cadherin and restores cell–cell adhesion, and concomitantly reduces E-Cadherin/PG complex formation [8,110]. Additionally, in cells overexpressing DSC2, several genes, such as AXIN2, SOX2, and TCF7, which inhibit the β-Catenin/TCF signalling pathway, are up-regulated [8], and NrCAM [111,112] and MMP-9 [113] are down-regulated.

### 4.2. Expression of DSG1 and DSG3 in ESCC

Studies have found that the localisation of DSG1 and DSG3 shifts from the cell membrane to the cytoplasm in ESCC. It has been suggested that this may either be due to the dissolution of desmosomes or reduced desmosome assembly. Lower levels of DSG1, DSG3, and desmosome expression cause the loosening of desmosome adhesion, and cells subsequently leave the primary tumour, leading to tumour invasion and metastasis [114]. An earlier study showed that there is a correlation between the lower expression of DSG1 (human protein) and tumour invasion, lymph node metastasis, and lymphatic invasion, and DSG1 may be a significant factor in the prognosis of ESCC [115]. In 2014, Fang et al. collected 162 specimens and performed immunohistochemical staining for DSG3. They found that DSG3 was not only expressed at high levels in ESCC but was also distributed abnormally. The high expression of DSG3 showed a significant correlation with regional lymph node metastasis; however, negative DSG3 expression contributed to a poor survival rate [116,117]. Such contradictive results need further research.

mRNA levels for PG and DSP positively correlated with DSG3 [117]; however, no additional studies have shown the relationship between DSG3 and ESCC. 

### 4.3. PG Loss and β-Catenin Accumulation in the Cross-Talk between Adherens Junctions and Desmosomes

Other components of the desmosome, such as PG, which is mentioned above in Section 4.1, and is also known as γ-Catenin, help maintain normal epithelial tissue structure [118,119]. PG is a tumour metastasis suppressor and can be used as an independent prognostic factor for survival. T-LAK cell-originated protein kinase (TOPK) interacts with PG through a TOPK-γ-Catenin binding complex to promote the invasion of ESCC cells by activating the Src/GSK3β/STAT3 and ERK signalling pathways via PG [120]. Findings show that GSK3β mediates the degradation of β-Catenin [77] and Snail [88,90], and the associated E-Cadherin is internalised through Src [64]; thus, PG plays a vital role in the cross-talk between adherens junctions and desmosomes, and therefore, it is generally seen as a component of both adherens junctions and desmosomes [119]. As PG is expressed at lower levels in ESCC, it is accompanied by the decrease of E-Cadherin and DSC2, thus resulting in reduced cell–cell adhesion, which leads to cell migration. PG and β-Catenin are members of the armadillo family of proteins [110]. As discussed above, with ESCC, lower DSC2 levels cause PG to compete with β-Catenin when binding to E-Cadherin, thus leading to the altered functioning of adherens junctions [8]. This indicates an unexplored area with regard to PG’s influence on the cross-talk that occurs between adherens junctions and desmosomes, and its clinical application.

Iwaya’s team tested the mRNA level of envoplakin (EVPL) in ESCC cell lines. Results indicated that although the EVPL is a strong candidate in terms of being the ESCC target gene according to its trait and localisation, it may not be the target gene for ESCC because experiments showed that it does not mutate; however, results also demonstrated that it is frequently deleted in ESCC [121]. Other cell–cell junction components that have not yet been studied in ESCC can also be explored. In a RIP1-Tag2 (RT2) mouse model of islet cell carcinogenesis, DSP has been shown to influence local tumour invasion, but it does not influence vast tumour invasion and metastasis [122]. PKP3 can also mediate the assembly of desmosomes and promote E-Cadherin maturation through Rap1 GTPase in squamous cell carcinoma (SCC) 9 cells because the lack of Pkp3 disrupts the E-Cadherin/Rap1 complex, which is a necessity for adherens junctions [123]. 

## 5. Gap Junctions

The gap junction is an intercellular channel structure encoded by a family of genes called connexins. Unlike other junctions, gap junctions do not prevent substances from passing between cells. Instead, gap junctions allow two adjacent cells to communicate through corresponding channels with connexins (Cxs), and they play an important role in cell communication. The channel comprises two membrane-integrated hemichannels that are supplied by both of the two adjacent cells. Each hemichannel includes a hexameric complex of Cxs proteins [115]. There are at least 21 Cx isoforms in the human genome [124]. Small ions and molecules (1000 Da) directly pass through gap junctions. Gap-junctional intercellular communication (GJIC) is thought to be involved in tissue homeostasis, cell differentiation, and cell growth [125,126].

Reduction or loss of gap junction activity is associated with various human cancers, including ESCC [127,128]; however, the mechanism of action in ESCC remains unknown, and there are only a limited number of studies related to Cx26 and Cx43 [129]. Cx26 and Cx43 are the Cxs constitutively expressed in normal epithelial oesophageal tissue, but in most oesophageal tumours, the expression of Cx26 is absent, and the expression of Cx43 is decreased [130,131]; however, during tumour progression and the acquisition of the malignant phenotype, Cx proteins often translocate from the cell membrane into an intracellular site such as cytoplasm, which causes the number of Cxs to increase in different histological types of malignant tumours [132], and the excessive accumulation of Cxs may be related to cancer progression [133]. A study analysed the expression of Cx43 via immunohistochemical staining and found that Cx43 is expressed at a high frequency in patients with ESCC. Moreover, in patients with high Cx43 expression, the survival rate is lower compared with those that have low Cx43 expression [134]. Another study found no positive staining for the specific expression of Cx26 in normal oesophageal epithelial cells, whereas positive Cx26 expression in tumours was correlated with lymph node metastasis and a low five-year survival rate in ESCC patients. This suggests that the abnormal expression of Cx26 participates in the progression of ESCC [135].

The mechanisms of whether and how Cxs behave in a pro-oncogenic manner are not yet clear; however, it was proposed that the endoplasmic reticulum stress (ER-stress) response may be closely connected with this process. Since endoplasmic reticulum (ER) is a critical organelle with functions that include protein folding and degradation, it is therefore vital to maintain homeostasis in all ER components and machineries [136]. The cancer cells often lead to conditions that promote the build-up of misfolded proteins. The accumulation of unfolded or misfolded proteins leads to stress conditions [137]. Then eukaryotic cells respond rapidly to ER dysfunction through a series of adaptive pathways called ER stress pathways [138], which then activate the unfolded protein response (UPR) in order to maintain ER homeostasis; however, if these processes fail to resolve ER stress, a terminal UPR program takes over and actively signals cell suicide [139]. Although many proteins related to the ER-stress response function in a Golgi-independent manner, the ER-resident ATF6 protein is translocated into the Golgi apparatus, where it is cleaved, and then imported into the nucleus to induce genes that participate in the ER-stress response [132,140]. In experiments on rat skin, translocation of Cx43 might be related to the activation of Wnt signalling, which also plays an important role in β-Catenin phosphorylation, and consequently, E- to N-Cadherin transition [141]. Studies of breast cancer cells found that N-Cadherin/E-Cadherin junctions and Cx43 participate in the composition of the osteogenic niche. This intermediate space allows heterotypic adherens junctions between E-Cadherin on disseminated tumour cells, and N-Cadherin on osteogenic cells, to stimulate mTOR signalling in cancer cells to support growth and metastasis [142,143]; therefore, it is worth exploring their relationship further to help understand the roles in ESCC.

Existing studies point to a model wherein the activity of gap junctions is reduced or lost in most ESCC tumours with a low degree of malignancy. Then, as cancer develops, the abnormal expression and increased intracellular Cx expression will occur in poorly differentiated ESCC cells, which is indicative of poor patient prognosis.

## 6. Therapeutic Implication

The cancer therapy for claudin abnormal expression mainly involves highly expressed claudin molecules combined with CPE to mediate tumour cell lysis, and they are treated as antigen targets or antibody coupling drugs [144]; however, for the most part, claudin is minimally expressed in ESCC. Studies have shown that the overexpression of histone deacetylases (HDACs) promotes ESCC [145,146,147]; therefore, HDAC inhibitors might be promising antitumour drugs for ESCC. Further research has been carried out on other tumours. Marked up-regulation of HDAC-4/ERK1/2/claudin-2 signalling has been demonstrated in colon cancer [147]. HDAC inhibitors suppress the proliferation, migration, and invasiveness of head and neck squamous cell carcinoma (HNSCC) by down-regulating the p63-mediated tight junction molecules JAM-A and claudin-1 [148]. Trichostatin A, an HDAC inhibitor, suppresses ESCC cell growth by activating the PI3K/Akt and ERK1/2 pathways [149]. Collectively, the up-regulated HDAC in ESCC may regulate claudin by up-regulating ERK1/2 activation to promote the progress of ESCC. This may play an important role in the exploration and understanding of the theoretical mechanism of HDAC inhibitors in the treatment of ESCC.

As for occludin, it has been proven that Raf1-induced tumour cells can rescue the epithelial phenotype and induce the reassembly of functional tight junctions by inducing exogenous occludin, which implies that the introduction of exogenous occludin may also apply to ESCC therapy for its low expressing occludin [150]. Moreover, a study found that a diosmectite-zinc oxide composite improves intestinal barrier restoration, in addition to increasing the expression of occludin, claudin-1, and ZO-1; therefore, diosmectite-zinc oxide also has the potential to become a drug that strengthens the oesophageal epithelial barrier while reducing the invasion and metastasis of ESCC [151].

E-Cadherin methylation is expected to be a new therapeutic target for the treatment of non-small cell lung cancer and oestrogen receptor-negative or HER2-negative breast cancer with an aggressive tumour biology [152,153]. Non-response to EGFR-targeted treatment such as cetuximab for colorectal cancers was associated with low E-Cadherin expression. Thus E-Cadherin expression could be a potential predictive and sensitive marker for EGFR-targeted therapy [154,155]. Although none of the β-Catenin inhibitors go beyond preclinical studies, strategies were proposed to develop the small molecule binding and depleting cellular β-catenin [156].

Various signalling pathways are involved in EMT, which relate to E-Cadherin and N-Cadherin switches. Multiple natural compounds with anticancer activity have been shown to inhibit EMT by suppressing key molecules or pathways such as N-Cadherin and increasing expressions within the E-cadherin/β-catenin complex [14]. Inhibitors of N-cadherin have anticancer therapeutic potential. ADH-1 has been studied in greater depth, and is capable of causing apoptosis and blocking angiogenesis [157]. Based on clinical trials thus far, ADH-1 might be useful in treating ovarian cancer [158], and it was given orphan drug status by the United States Food and Drug Administration for the treatment of melanoma in 2008 [159]. Such strategies are promising for the development of ESCC treatments.

There is a paucity of research on the use of desmosomes in targeted therapies for various diseases. Although there are currently not a great many drugs using desmosomes as targets, we can focus on not only the disease markers themselves but also their upstream and downstream impacts when studying the targets of targeted therapies. Studying MiR-25-mediated tumorigenesis for the treatment of oesophageal cancer may be a desirable research direction [160].

A study reported that Cx43 significantly increased glioblastoma cell sensitivity to paclitaxel and doxorubicin [161]; therefore, a high expression of Cx43 may be exploited with the delivery of the targeted drug to cancer cells via enhanced drug permeability. However, the expression of Cx43 in ESCC can act as a tumour suppressor or oncogene depending on the malignancy of the tumour; thus, if Cx43-targeted therapy progresses into clinic use, careful quantitative biomarker analysis and a better understanding of how Cx43 mediates cancer phenotypes in different contexts will be required [162].

## 7. Summary

Cell–cell junctions play essential roles in invasion, migration, lymph node metastasis, differentiation, and prognostic conditions in ESCC. The current literature shows that the altered localisation and the variation in expression level may promote the invasion and metastasis of cancer cells (Table 1). The mis-localisation of these proteins weakens cell-to-cell connection, thus enhancing the possibility of cell metastasis and aggressiveness. This mis-localisation from the cell membrane to the cytoplasm and perinuclear region is possibly connected with the specific signalling pathways or posttranscriptional modification; for example, the transfer of Claudin-1 expression from the cell membrane to the nucleus is associated with the activation of the Akt signalling pathway [26]. The cytoplasmic localisation of E-Cadherin may be caused by reduced glycosylation or the protein truncation of E-Cadherin [50]. This glycosylation prevents the proteins from adhering to the membrane but remains in the cytoplasm or perinuclear region. The methylation or phosphorylation modifications of adhesion-associated molecules, such as E-Cadherin, EphA3, and β-Catenin, may impact the activation of signalling pathways, which involves the transduction of signalling, transcription regulation, and apoptosis, thus affecting the occurrence and development of ESCC [51,59,80].

Beyond this, some molecules which are not components of cell–cell junction structures can also make changes to ESCC by affecting the structure of the cell junctions, such as the co-localisation of PAR-3 with ZO-1 participating in the formation of tight junctions [39]. Moreover, few studies are related to the role of adaptor proteins in the four cell–cell junctions in ESCC and can thus be a future area of study.

**Figure 3:**
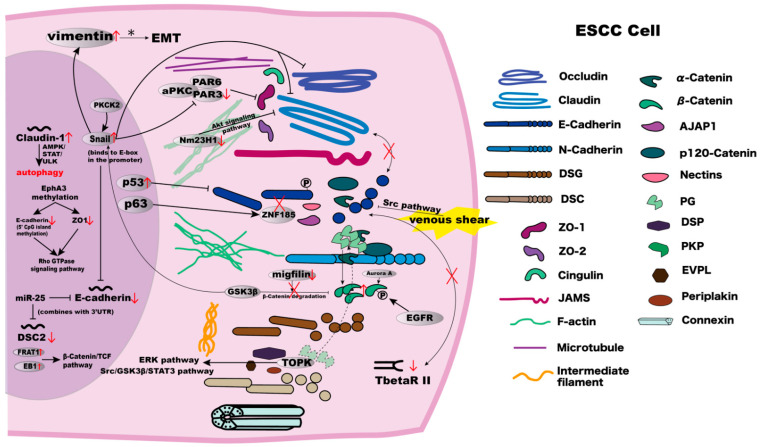
The mechanisms and interrelationships in components involved in cell–cell junctions in ESCC cells. Detailed explanations are summarised in Table 1.

The role of various cell junctions is not necessarily independent; the molecules in different cell–cell junctions may interact with each other (Figure 3). Overall, we concluded that cross-talks between adherens junctions, tight junctions, and desmosomes exist. Some adhesion molecules enhance tumorigenicity, and some suppress tumorigenicity. If the adhesion molecules that enhance tumorigenicity are inhibited, and those that suppress tumorigenicity are enhanced, will the activity of the tumour cells be inhibited? Knowledge of heterotypic interactions, carbohydrate epitopes, functions of intracellular localisation, and so on, is required when testing this hypothesis. If successful, these will be exciting findings to help medical treatment in the future. This is one such research direction that can be pursued in this area.

Moreover, it is worth noting that recent research indicates that some molecules in cell–cell junction structures have little effect on primary tumour initiation and growth, but they are instead critical for the formation of distant metastases, such as paxillin in breast cancer [163]. The function of claudin-11 and E-Cadherin in the collective migration for tumour progression has been reported [35]. There may be a reduced quantity of some molecules, or they may appear with an intracellular abnormal distribution, which impairs its adherence ability. It may cause cells to leave the primary tumour, leading to tumour invasion and metastasis; however, for spreading tumour cells such as circulating tumour cells (CTC), these adhesion-related molecules may be helpful for tumour cell survival. This theory has been partly supported in breast cancer, because a study found that the down-regulation of DSG2 in hypoxic tumours allowed for single tumour cell dissemination, whereas DSG2-expressing tumours generated more CTC clusters; therefore, when considering target treatments, we should try to avoid antibody internalisation and the enhancement of intravasation by deregulating the target molecules [3].

## 8. Conclusions

By reviewing the changes to the components related to tight junctions, adherens junctions, desmosomes, and gap junctions in ESCC, and the cross-talks between them, we hope that these descriptions can help readers further understand the role of cell–cell junction structure molecules in ESCC, explore specific targets for clinical examination and treatment, and provide practical ideas for early diagnosis and therapy for the disease.

## Figures and Tables

**Table 1:** Summary of changes and mechanisms of cell-cell junction components in ESCC.

Structure	Transmembrane Proteins	Adaptor Proteins	Expression	Consequence		Mechanism	Reference
Tight junction	Claudin-1	/	Membrane (↓)	Associated with recurrence and poor prognosis	Enhanced invasiveness	AKT signalling pathway, activated by reduction of Nm23H1; binding of Snail to E-box elements	[26,30]
			Nucleus (↑)		Enhanced proliferation and metastasis	Autophagy triggered by the AMPK/STAT1/ULK1 signalling pathway	[27]
	Claudin-4	/	Membrane (↓)	Associated with poor differentiation, deeper invasion, positive lymph node metastasis, low disease-free survival, and overall survival; independent risk factor for recurrence		Promoter methylation	[9,28]
	Claudin-7	/	Membrane (↓)	Associated with deeper invasion, advanced tumour stage, positive lymph node metastasis, and lymphatic invasion		Binding of Snail to E-box elements; loss of synergistic effect between claudin-7 and E-Cadherin	[12,24,30]
	Occludin	/	Membrane (↓)	/		Binding of Snail to E-box elements	[30]
	/	ZO1	Membrane (↓)	Associated with positive lymph node metastasis and poor differentiation		Decrease of PAR-3 (GSK-3β/Snail/Par3/ZO-1 axis); EphA3 methylation	[39,58]
Adherens junction	E-Cadherin	/	Membrane (↓)	Associated with the differentiation and prognosis	/	Methylation of CpG island; p53 mutation	[53,55,56,57,60]
					Promote proliferation and invasion	Down-regulation of claudin-7 causes loss of synergistic effect between claudin-7 and E-Cadherin	[12]
					Promote migration and invasion	EphA3 methylation, activation of the Rho GTPase signalling pathway; absence of TbetaRII expression	[49,50,59]
						High expression of miR-25 inhibits E-Cadherin expression which is crucial for mediating EMT	[59]
					Acquire resistance to anoikis and anoxia	High-activity of PKCK2, stabilisation of Snail, upregulation of N-Cadherin	[88,89,90,94]
				Destroy the integrity of the epithelium		Mutation of p63 and absence of the expression of ZNF185	[63]
			Cytoplasm (↑)	Associated with poor differentiation		Reduce the glycosylation of E-Cadherin or truncate the E-Cadherin	[50]
	N-Cadherin	/	Membrane (↑)	Induce a migratory phenotype, influence the ability of tumour cells by altering their cadherin expression profiles		High activity of PKCK2, directly and indirectly stabilises Snail (see Figure 2)	[88]
	/	α-Catenin	Membrane (↓)	Associated with an aggressive phenotype and lymph node metastasis		/	[71]
				Associated with tumour progression and poor prognosis		/	[70,72]
		β-Catenin	Cytoplasm (↑) Nucleus (↑)	Associated with poor prognosis, tumour cell invasion and metastasis	Dissociation from cell–cell contacts, intracellular accumulation, inhibition of E-Cadherin mediated cell adhesion	Phosphorylation of β-Catenin by EGFR, disrupting the binding between β-Catenin and α-Catenin	[80,83]
						FRAT1 and EB1 activating the β-Catenin/TCF pathway, promoting transcriptional activity of β-Catenin	[75,76]
						Down-regulation of migfilin, inhibiting the GSK-3β-dependent pathway, β-Catenin degradation and β-Catenin TCF-mediated transcriptional activity	[77]
						Overexpression of Aurora-A inhibiting degradation, increasing phosphorylated β-Catenin at Ser552 and Ser675	[79]
		p120-Catenin	Membrane (↓)	Associated with lymph node metastasis	Positively correlated with differentiation	/	[67]
Desmosome	DSG1	/	↓	Associated with tumour invasion, lymph node metastasis and lymphatic invasion		/	[115]
	DSG3	/		Associated with lymph node metastasis, but negative DSG3 expression contributed to a poor survival rate		/	[114]
	DSC2	/	Membrane (↓), Cytoplasm (↑)	Promote the invasion and metastasis		High expression of miR-25; binding of miR-25 to the DSC2 mRNA 3′UTR	[8,108]
			↓			Increased free PG (γ-Catenin), which competes with the β-Catenin in the E-Cadherin/β-Catenin complex and causes an increase in β-Catenin cytoplasmic delivery, nuclear localisation, and transcriptional activity; activating β-Catenin/TCF signalling pathway	[8]
		PG	↓	Cause reduced cell-cell adhesion and enhance cell migration		A tumour metastasis suppressor; lower DSC2 levels cause PG competes with β-Catenin to bind to E-Cadherin	[8,110]
Gap junction	Cx26; Cx43	/	Membrane (↓) Cytoplasm (↑)	Associated with an aggressive phenotype and lymph node metastasis		/	[131,133,134,135]

## Abbreviations

ZO	zonula occludens
TAMP	tight junction-associated MARVEL proteins
AJAP1	Adherens junctions-associated protein-1
Cdh23	cadherin-23
circAMOTL1L	angiomotin-like 1-derived circRNA
CTC	circulating tumour cells
Cx	connexin
DSC	desmocollin
DSG	desmoglein
DSP	desmoplakin
EGFR	epidermal growth factor receptor
EMT	epithelial-to-mesenchymal transition
ER	endoplasmic reticulum
ESCC	Oesophageal squamous cell carcinoma
EVPL	envoplakin
GSK-3β	glycogen synthase kinase-3β
GUK	guanylate kinase
HDACs	histone deacetylases
HSP70RY	heat shock 70kD protein 4
JAMs	junction adhesion molecules
LPS	lipopolysaccharide
miR-25	microRNA25
Nm23H1	non-metastatic protein 23-H1
PCDH10	protocadherin 10
PDZ	PSD95, DlgA and ZO-1
PG	plakoglobin
PG	plakoglobin
Pkp	plakophilin
RBM25	RNA Binding Motif Protein 25
SCC	squamous cell carcinoma
SH3	SRC homology 3
TOPK	T-LAK cell-originated protein kinase
UPR	unfolded protein response
ZNF185	zinc finger protein 185
ZONAB	ZO-1-associated nucleic acid binding protein
